# Druggable Pockets at the RNA Interface Region of Influenza A Virus NS1 Protein Are Conserved across Sequence Variants from Distinct Subtypes

**DOI:** 10.3390/biom13010064

**Published:** 2022-12-29

**Authors:** Sarah Naceri, Daniel Marc, Rachel Blot, Delphine Flatters, Anne-Claude Camproux

**Affiliations:** 1 Unité de Biologie Fonctionnelle et Adaptative, CNRS, INSERM, Université Paris Cité, F-75013 Paris, France; 2 Equipe 3IMo, UMR1282 Infectiologie et Santé Publique, INRAE, F-37380 Nouzilly, France; 3 UMR1282 Infectiologie et Santé Publique, Université de Tours, F-37000 Tours, France

**Keywords:** binding site, influenza A virus, non-structural protein 1, groove-pocket, drug design, structural polymorphism

## Abstract

Influenza A viruses still represent a major health issue, for both humans and animals. One of the main viral proteins of interest to target is the NS1 protein, which counters the host immune response and promotes viral replication. NS1 is a homodimer composed of a dimeric RNA-binding domain (RBD), which is structurally stable and conserved in sequence, and two effector domains that are tethered to the RBD by linker regions. This linker flexibility leads to NS1 polymorphism and can therefore exhibit different forms. Previously, we identified a putative drug-binding site, located in the RBD interface in a crystal structure of NS1. This pocket could be targeted to block RNA binding and inhibit NS1 activities. The objective of the present study is to confirm the presence of this druggable site, whatever the sequence variants, in order to develop a universal therapeutic compound that is insensitive to sequence variations and structural flexibility. Using a set of four NS1 full-length structures, we combined different bioinformatics approaches such as pocket tracking along molecular dynamics simulations, druggability prediction and classification. This protocol successfully confirmed a frequent large binding-site that is highly druggable and shared by different NS1 forms, which is promising for developing a robust NS1-targeted therapy.

## 1. Introduction

Influenza A viruses are enveloped viruses belonging to the family Orthomyxoviridae [1]. They infect a wide spectrum of bird and mammal species, and in humans they are responsible for seasonal epidemics and global pandemics, resulting in severe pneumonia and death in affected patients. The 1918 influenza pandemic virus caused severe pneumonia, resulting in an estimated 50 million deaths worldwide. The morbidity and mortality rate of the influenza A virus reaches three to five million severe cases and 290,000 to 650,000 deaths each year [2,3,4]. Currently, there are few alternatives to the influenza vaccine, the efficacy of which depends on the subtype of influenza A virus and can be jeopardized by amino-acid substitutions in hemagglutinin, the major viral antigen.

Small compound therapy can be useful when prevention measures do not suffice. Currently, the available anti-influenza drugs comprise the neuraminidase and the viral polymerase inhibitors, both of which were designed based on the knowledge of target proteins structures. Non-structural protein 1 (NS1) stands as a possible additional target for novel antiviral compounds. NS1 has been extensively studied, because of its multifunctional character. Absent from the viral particle, it is highly expressed in the cytoplasm and nucleus of infected cells, where it is able to interact with various components involved in the interferon system, inhibiting this response. NS1 relies on several mechanisms to attenuate the interferon response during influenza-virus infection, and stands as the main interferon antagonist encoded by the virus [5]. This prominent role and its requirement to promote viral replication [6] make it a promising target for novel antiviral therapies. Thus, targeting the NS1 protein may provide a good outcome in the treatment of influenza infection [7,8,9].

NS1, a homodimer of two 230 residue polypeptides, is comprised of three structural domains, where only residues 1–203 have been resolved in crystallography. The RNA-binding domain (RBD) is an obligate dimer of residues 1–73, which is connected to the two effector domains (ED, residues 86–203) by the short, unstructured, linker regions [10]. The RBD is arranged as three pairs of symmetrically positioned antiparallel alpha-helices. Residues 86–203 of each chain fold autonomously into an ED. To explore the NS1 protein as a target for drug design, three dimensional (3D) structures of the protein are needed. However, only four full-length NS1 proteins (from subtypes H6N6, H1N1 and H5N1) have been crystallized and are available as 3D structures in the Protein Data Bank (PDB) [11]. The flexible linker enables the quaternary structure to adopt polymorphic shapes, of which three main forms have been observed: closed, semi-open and open, according to the position of each ED relative to its counterpart and to the dimeric RBD [12,13,14].

B.G Hale reviewed the conformational plasticity of NS1 [13]. This study underlined the importance of considering the dynamic properties of NS1, essential for its multiple functions. Therefore, NS1 conformational plasticity can impact the structure. Using computational molecular-dynamics (MD) simulations, our team explored the dynamic and flexibility properties of the four above-mentioned full-length NS1 structures [15]. Molecular-dynamics simulation is a computational approach classically used to characterize the variability of the global domain or 3D structure. In order to explore to what extent the structure as well as its flexible conformation were robust to variations in the amino-acid sequences, the four structures were submitted to MD simulation, allowing us to follow the dynamic of the conformational changes over time. The gradual switch from closed to open form along the MD simulations was studied by computing the three distances between the centers of mass of the domains and, notably, between the ED monomers, to characterize the opening state of the conformation. Our results confirmed the co-existence, regardless of the subtypes, of three structural forms in a dynamic equilibrium. Our data also suggested that the linker length, as well as the presence of specific amino acids, modulate the dynamic properties and the flexibility of NS1. Finally we confirmed the remarkable stability of the RBD for different subtypes, regardless of the forms and linker size, contrary to the ED, thus emphasizing the interest of targeting the RBD using drug-design approaches to develop a strain-independent therapy [15].

Other studies [16] have performed pocket extraction and druggability prediction on several PDB-registered crystal structures, focusing on ED monomers or ED dimers in different forms. Three main druggable pockets were thus identified in the ED, corresponding to regions that are highly conserved across different virus subtypes. Combining sequence analysis and druggability-prediction algorithms, the same authors [17] systematically probed in silico the druggability of the NS1 effector domain. Given the flexibility of the ED domain and its interaction with many partners, it proved more relevant to target the RBD for possible therapy because, beyond its stability, it is the main partner of the RNA leading to viral replication through its interaction.

To explore NS1 as a therapeutic target, studies have been carried out by researchers who have hypothesized that inhibiting NS1-RBD interaction could alter the functionality of the RBD and significantly reduce the replication potential of the virus and its pathogenicity [18,19]. This work showed that the RBD interface region is rather well conserved among influenza A viruses, based on PDB-registered crystal structures. They described a deep pocket, localized between the antiparallel alpha-helices 2 and 2′ of the RBD interface, which could be explored as a potential drug-binding site of NS1. However, many studies have been developed and have confirmed the importance of predicting the druggability of proteins and binding sites in order to prioritize the targets for the development of therapies [20].

Although the RBD was confirmed as a very stable dimer [15], there is an inherent structural flexibility that could impact the pocket druggability. Indeed, the importance of taking into account the cryptic or hidden, transient and flexible pocket to identify reliable binding sites for drug design is increasingly highlighted [21]. Recently, our group preliminarily confirmed interest in considering the flexibility of the 3D structure using molecular dynamics simulations to extract druggable pockets from the RBD in the case of the H6N6 NS1 subtype with a structure in a closed form [22]. We performed a pocket analysis by considering the intrinsic flexibility of NS1, using H6N6 NS1 full-length protein MD simulations. Our main result was the identification of a druggable binding site located between the two antiparallel helices α2 and α2′ forming the RNA-binding interface, which we defined as the “RBD-groove”. While this RBD-groove was not detected as a druggable pocket in the rigid crystal structure, its druggable properties were consistently observed in a substantial fraction of the conformations that were sampled during the MD simulations. This frequently observed druggable pocket confirmed the potentiality to prevent the binding of NS1 to RNA.

In this study, our aim is to confirm that such a druggable RBD-groove is stable, by considering NS1 structural polymorphism, to validate the interest of targeting the RBD for developing a robust and subtype-independent small compound targeting NS1. To that end, we study the existence of a druggable binding site located at the RBD-groove by considering its structural flexibility and NS1 structural polymorphism (shape and linker size). Our approach consists in estimating pockets conformation sampled from MD simulations on four available full-length NS1 structures from three subtypes (H6N6, H1N1, H5N1). We focused on pockets located at the RBD-groove, and characterized their druggability properties. A classification of these pockets based on residue-composition similarity allowed us to identify three main druggable clusters of groove-pockets describing putative druggable binding sites. These latter were analyzed in terms of frequency, residue composition, physico-chemical and druggability properties, variability and representativeness of different subtypes of the influenza A virus. One of these sites is of particular interest to target by the drug-design approach, as it is a large, druggable site, stable to structural polymorphism, and therefore common to the different subtypes.

## 2. Materials and Methods

### 2.1. Selection of NS1 Full-Length Structures and Study of Their Flexibility

#### 2.1.1. Selection of Four Different Forms of the NS1 Protein

Three NS1 proteins from subtypes (H6N6, H1N1, H5N1) are considered, co-crystallized in full-length and available as 3D structures in the PDB. Although RBD has been shown to be stable, it is important to perform the full-length protein study to assess the impact of the NS1 polymorphism during pockets tracking.

**Figure 1:**
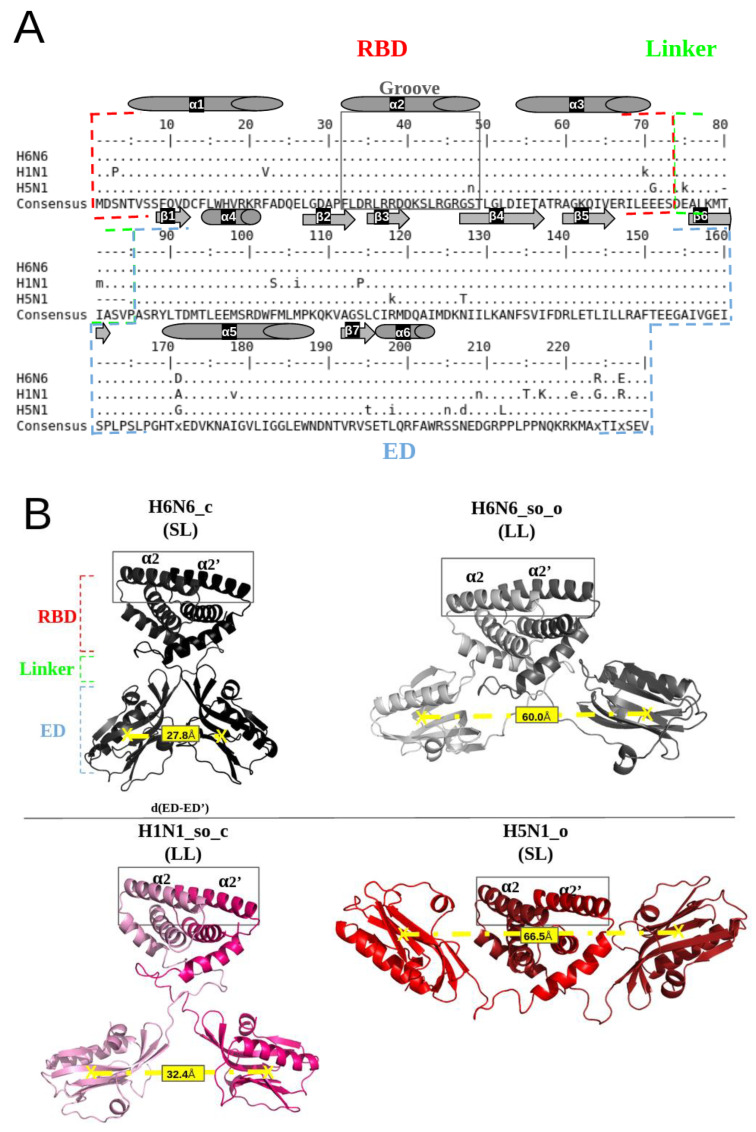
Illustration of the NS1 sequence conservation and the structural polymorphism of the NS1 protein: (**A**) Alignment of H6N6, H1N1 and H5N1 sequences extracted from the UniProt database (accession number: P03496, A5A5U1, Q20NS3 respectively) [25] using the “showalign” tool of software suite EMBOSS [24], showing conservation of the RBD region and particularly the “RBD-groove”, where the sequence is boxed in full gray lines. The RBD, ED and linker (long (LL) or short (SL)) are bordered by red, blue and green dotted lines, respectively. The α-helices are represented by cylinders and the β-sheets by arrows. Only differences relative to the consensus sequence (above and underneath) are shown, and the consensus line can be displayed in a mixture of upper- and lower-case symbols. Upper case indicates strong consensus and lower case indicates weak consensus; hyphens in H5N1 sequence are deletions (Δ80–84). (**B**) Representation of the four NS1 initial structures (4OPA, 4OPH, 5NT2 and 3F5T), colored in black, gray, pink and red, respectively, using the PyMol visualization software [26]. The “RBD-groove” is bordered by gray, full lines enclosing the two α2 and α2′ helices. The boundaries of the RBD, ED and the linker are shown in red, blue and green, respectively. The d(ED-ED’) distance between the EDs monomers centers of mass quantifies the structural polymorphism of the different structures, which is on average 27.8 Å for H6N6_c, 60.0 Å for H6N6_so_o, 32.4 Å for H1N1_so_c and 66.5 Å for H5N1_o.

Figure 1A illustrates a multiple sequence alignment, calculated with the ClustalΩ algorithm [23], of H6N6, H1N1 and H5N1 sequences from the UniProt database (Table S1), using the EMBOSS tools [24]. It can be noted that, despite sequence mutability, there is strong sequence conservation at the α2 and α2′ helices in the groove region. The RBD (1–73 amino acids) and ED (86–203 amino acids) domains connected by short (SL) or long (LL) linker regions (deletion or not of five residues “Δ80–84”) are indicated in Figure 1B.

Four full-length NS1 crystallographic structures associated with three different subtypes of H6N6 (pdb codes: 4OPA and 4OPH), H1N1 (pdb code: 5NT2) and H5N1 (pdb code: 3F5T) are currently available in the PDB and were considered in this study [12,27,28]. After the rebuilding and reversion of the engineered mutations, i.e., R38A/K41A, and specifically the W187A mutation for 5NT2, the complete conformations of the initial structures’ templates were obtained. The corresponding four initial structures are described in Table S1. The PDB structures 4OPA and 4OPH correspond to the H6N6 subtype but are in different forms and linker sizes: 4OPA is a closed form (its ED are separated by a distance of 28 Å) associated with a short linker (five amino acids Δ80–84 are deleted), whereas 4OPH is a semi-open form with open tendency (its two ED are separated by a distance of 60 Å) (Table S1), associated with a long linker (for distance measurement between two EDs, see Naceri et al., 2022 [15]. They are named H6N6_c and H6N6_so_o. The 5NT2 structure corresponds to the H1N1 subtype in a homodimeric structure and is classified as a semi-open form with closed tendency (its two ED are separated by a distance of 47 Å,) associated with a long linker, named H1N1_so_c. The 3F5T corresponds to the H5N1 subtype in an open form (its EDs are separated by a distance of 69 Å), associated with a short linker (Δ80–84), named H5N1_o.

These four initial NS1 structures (H6N6_c, H6N6_so_o, H1N1_so_c and H5N1_o) are illustrated in Figure 1B, with the RBD-groove indicated, as defined by Abi Hussein et al., 2020, and corresponding to the region between the two antiparallel helices α2 and α2′ forming the RNA-binding interface [22].

#### 2.1.2. Sampling of NS1 Conformations during MD Simulation

MD simulations were run for 150 ns, in an isothermal–isobaric (NPT) ensemble at 300 K and 1 bar, at pH 7 and using the AMBER-99SB force field with the GROMACS software package V2019.5 [29]. The detailed protocol is described in Naceri et al., 2022 [15].

The sample of 500 frames of the NS1 full-length conformations were stored at regular intervals from each of the four MD simulations generated from the crystallographic structures (H6N6_c, H6N6_so_o, H1N1_so_c and H5N1_o). A resulting set of 2000 NS1 full-length conformations was obtained, with 500 conformations extracted from MD simulations obtained on each structure.

### 2.2. Machine-Learning Analysis of the Groove-Pockets to Identify Main Pocket Cluster

#### 2.2.1. Estimation of Groove-Pockets

From these 2000 NS1 conformations, pocket extraction and characterization were performed using the PockDrug tool preliminary developed by the team [20]. Pockets were estimated using an automated geometry-based Fpocket [30]. Estimated pockets were characterized in terms of 19 physicochemical, geometrical properties and a druggability score prediction [31]. The list of residues involved in each pocket was also additionally extracted.

In this study, we applied three different filters (size of pocket ≥ 8 residues, pockets located on the groove and only druggable ones) to select pockets. The pockets considered in the groove are those including at least one residue from each of the two α2 and α2′ helices located on each RBD monomer of the NS1 protein (residues 30–50) that delimit the groove, as proposed by Abi Hussein et al., 2020 [22]. The corresponding pockets are called “groove-pockets”. The last filter to select druggable pockets using the PockDrug is a threshold druggability score (≥ 50%) indicating a druggable pocket.

#### 2.2.2. Identification of Main Clusters of Pockets Located at the Groove Region

In Abi Hussein et al., 2020, we studied the evolution and similarity of the pockets along MD simulations in terms of the Euclidean distance between the pockets from PockDrug, based on the physico-chemical and geometrical descriptors [22]. In this work, we aim to study the evolution and similarity of pockets for three NS1 distinct sequence variants, H1N1, H6N1 and H5N1, and the impact of structural polymorphism on the occurrence of pockets and their druggability. Thus, we decided to quantify the pockets similarity in terms of common residues, using binary distance. A binary distance of 0 corresponds to two pockets including identical residues, and a distance of 1 corresponds to two pockets without common residue. We performed a hierarchical classification of the pockets using the binary distance using the Ward metric method (ward.D2) [32,33] and the R Hclust package [34].

Dendrogram visualizations were performed using the Heatmaps2 package in R (v 3.9) [35] to illustrate pocket similarity in terms of residues. The difference in terms of residues between pockets and pocket clusters increases with the dendogram branch-lengths (representative of the average binary distances). The resulting classification visualizes the similarity of the pockets in terms of common residues, and allows the identification of different main clusters of similar pockets.

### 2.3. Analysis of Druggable Binding Sites Identified by Main Pocket Clusters

Pockets with a similar residue composition are clustered within the same cluster, while pockets with different residue compositions are divided among different clusters. In this way, the set of similar pockets in a cluster can be considered as describing the flexibility of an associated binding site during the MD simulations for the four NS1 structures (H6N6_c, H6N6_so_o, H1N1_so_c and H5N1_o). The number of pockets in a cluster indicates the frequency of appearance of the binding site on the 2000 RBD-groove conformations. It provides quantification of the persistence of this binding site when considering groove evolution over time. The flexibility of the binding site is described by the residue variability of its associated pocket cluster. Highly frequent residues of a binding site (observed in more than 75% of the pocket cluster) are considered as a base, and named key-residues.

The analysis of main pocket clusters allows the detection of recurrent druggable binding sites and their characterization in terms of appearance frequency, key-residue composition, variability and druggability properties. Each binding site can be described by the analysis of its cluster of pockets. The size of the cluster provides the occurrence of a binding site along the MD simulations. The variability analysis of the pocket residues allows for the studying of the deformability of its corresponding site. The most frequent residues of the pocket cluster are considered as key residues of the binding site.

The main pocket clusters can also be analyzed in terms of the composition of the four structures (H6N6_c, H6N6_so_o H1N1_so_c and H5N1_o). Thus, we can determine if a binding site is specific, or common to the four considered structures.

## 3. Results

### 3.1. Extraction of Pockets Located at the RBD-Groove and Analysis of Its Druggability

The druggability of the RBD-groove for the four initial structures (H6N6_c, H6N6_so_o, H1N1_so_c and H5N1_o) was first analyzed. A groove was considered druggable if it included at least one druggable pocket. We searched the pockets located in the groove region, i.e., between helices α2 and α2′, and characterized them in terms of druggability, using PockDrug. For three out of the four initial structures, we observed between one and three pockets in the RBD-groove that are predominantly non-druggable; see Table 1. However, two pockets in the RBD-groove of H1N1_so_c are predicted to be druggable by PockDrug (with druggability scores of 0.57 and 0.68), indicating that the H1N1_so_c RBD-groove is druggable. Therefore, considering the four initial crystal structures of NS1, only a quarter of the RBD-grooves can be identified as druggable.

The evolution of the RBD-groove in terms of druggability was then explored by molecular dynamic simulations, to consider the flexibility of the structures. We analyzed the 2000 conformations, corresponding to four sets of 500 conformations extracted from each MD simulation of H6N6_c, H6N6_so_o, H1N1_so_c and H5N1_o (see method section).

We observed a total of 3360 pockets located at the RBD-groove and extracted from the 2000 conformations. This results in more than one pocket per RBD-groove (1.68 on average) for each conformation. Furthermore, a homogeneous distribution of these pockets was observed, ranging from 740 (22%) to 933 (28%) on the four samples of 500 conformations obtained from H6N6_c, H6N6_so_o, H1N1_so_c and H5N1_o (Table 1).

Overall, we observed that almost 40% (1272/3360) of the groove-pockets were druggable. Although the average number of pockets in the groove was almost equivalent for the four simulations, a more marked difference was observed with respect to their druggable properties: only 30% and 35% of the observed pockets were druggable for H6N6_c and H1N1_so_c, respectively, compared to almost 40% and 50% for H6N6_so_o and H5N1_o, respectively.

We then focused our analysis on the druggable RBD-grooves, i.e., on the 55.1% of the conformations exhibiting an RBD-groove that had at least one druggable pocket. This consistently observed druggability of the RBD-groove during the MD simulations is confirmed here for the four considered structures. Almost half of the RBD-grooves are druggable on the closed form (H6N6_c) and the semi-open form with closed tendency (H1N1_so_c); see Table 1. Concerning H6N6_c, the frequency of the druggable RBD-groove (47.8%) is very close to the one (~44%) obtained by Abi Hussein et al., 2020, which used three short MD simulations of 50 ns each, and only took into account pockets with more than 14 residues [22]. The higher frequency of druggable RBD-grooves (69.0%) is observed on the H5N1_o, leading to the suggestion that the open form may be more suitable for the presence of druggable pockets. Indeed, the second highest frequency of druggable RBD-groove (57%) is observed on the H6N6_so_o, in the semi-open form close to an open form.

Our results confirmed the importance of MD simulations in studying pockets druggability. Indeed, the groove-pockets are detected as druggable in more than half of the conformations over time, for the NS1 protein from different subtypes.

Consequently, the RBD-groove region meets the druggability criteria in 55.1% of the conformations for the different NS1 subtypes. We can note that the number of druggable pockets increases with the opening of the structure; the structure which presents the fewest druggable pockets is the H6N6_c, and the highest one is the H5N1_o, wherease semi-open forms presents a different number of druggable pockets depending on their degree of opening and closing.

This confirms that the RBD-groove region is able to form potential binding pockets, which, in more than half of the conformations, meet the druggability criteria, during MD simulations of the four initial structures. This groove region is an interesting candidate for targeting by a drug-like ligand for the different structures, regardless of its polymorphism.

### 3.2. Identification of Main Clusters of Druggable Groove-Pockets on Different NS1 Subtypes

**Figure 2:**
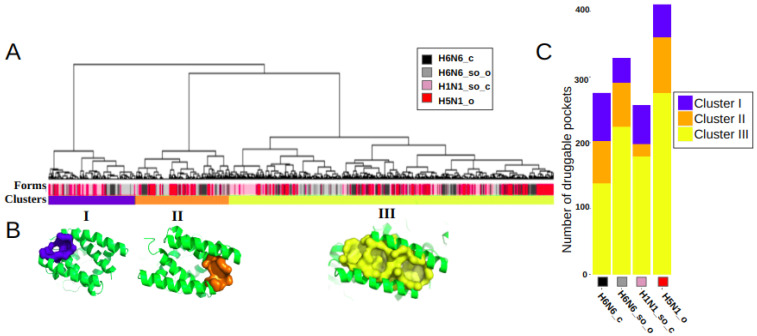
Classification and representation of the three main identified druggable groove-pocket clusters: (**A**) Hierarchical classification of druggable groove-pockets extracted from the MD simulations of the four crystallographic NS1 structures (H6N6_c, H6N6_so_o, H1N1_so_c and H5N1_o), indicated by the colors black, gray, pink and red, respectively, on the Forms line. Three clusters (I, II and III) were identified in the *Clusters* line, colored blue, orange and yellow, and comprising 220, 236 and 816 druggable groove-pockets respectively. (**B**) Representative pockets of three binding sites are illustrated on one representative groove conformation, in blue, orange and yellow for clusters I, II and III, respectively. (**C**) Histogram of the distribution of druggable pockets per cluster for each of the H6N6_c, H6N6_so_o, H1N1_so_c and H5N1_o structures.

We performed a hierarchical clustering of these 1272 druggable pockets, based on their residues composition, to identify main clusters and quantify their frequency of occurrence in the four different NS1 structures. Figure 2A illustrates the classification, resulting in three main clusters, I, II and III. These three most frequently observed clusters correspond to three distinct binding sites, noted as I, II and III, with a frequency of occurrence of 17.3%, 18.5% and 64.1%, respectively, of the 1272 druggable groove-pockets.

The clusters are well differentiated, as illustrated by the long length of the branches (proportional to the distance between them), indicating the absence or scarcity of common residues. On the other hand, pockets within a cluster are separated by very small distances, indicating that they are composed mainly of similar residues. This proximity of intra-cluster pockets confirms that the pockets of a cluster correspond to a same binding site. The variability of the pockets of a cluster in terms of residues allow the description of the flexibility of the binding site along the dynamics. The heatmap in Figure S1 presents the classification of the pockets and their associated residues. It shows, for instance, no common residue between clusters I and II. One pocket of each of these three clusters is represented in Figure 2B. This figure illustrates how pockets associated with clusters I and II are located at the opposite side of the groove; however, the three clusters are observed in the four structures. The histogram in Figure 2C shows the equilibrium representation of the three binding sites in a quantified way on the four distinct structures. However, we can observe a higher frequency of binding sites I and II in the closed form H6N6_c and a higher frequency of the binding site III in the open or semi-open-tending-to-open form (H5N1_o and H6N6_so_o).

As these three binding sites share few common key residues, some site co-occurrence is possible within a groove conformation. However, we found that the majority (85.4%) of the 1102 druggable RBD-grooves included only one of these druggable binding sites (on the same sampled conformation), with a similar frequency for the four NS1 structures. Two concomitant binding sites were observed in only 13.9% of the time-sampled druggable RBD-grooves, while 0.7% of them simultaneously exhibited one druggable pocket in each of the three sites (for an example, see Figure S2).

The three binding sites are observed on the four structures, as illustrated on Figure 2B. Thus, it can be concluded that three identified druggable binding sites are similarly observed on the four NS1 subtypes. These three sites appear repeatedly and frequently throughout the 150ns MD simulations obtained from the four different NS1 structures considered. Pockets are regularly observed along the MD simulations, as illustrated in Figure S3, on a sample of 100 conformations of each trajectory.

### 3.3. Characterization of Druggable Binding Sites of the Groove

Each binding site can be described through the analysis of its associated cluster of pockets. The size of the cluster provides the occurrence of a binding site on the 2000 conformations. The binding sites I and II are observed in at least 17.3%, while the most frequent binding site, III, is observed in 64.1% of the 1272 druggable groove-pockets, i.e., in 40% of the time-sampled conformations (Table 1). Binding sites I and II correspond to rather small pockets, and they include, on average, 11.7 ± 2.6 and 13.3 ± 4.9 residues per pocket, associated with an average druggable score of 66% ± 12 and 68% ± 13, respectively. The most frequent binding site, III, corresponds to larger pockets of 31 ± 7.1 residues on average, associated with an average druggability score of 71% ± 13.

**Figure 3:**
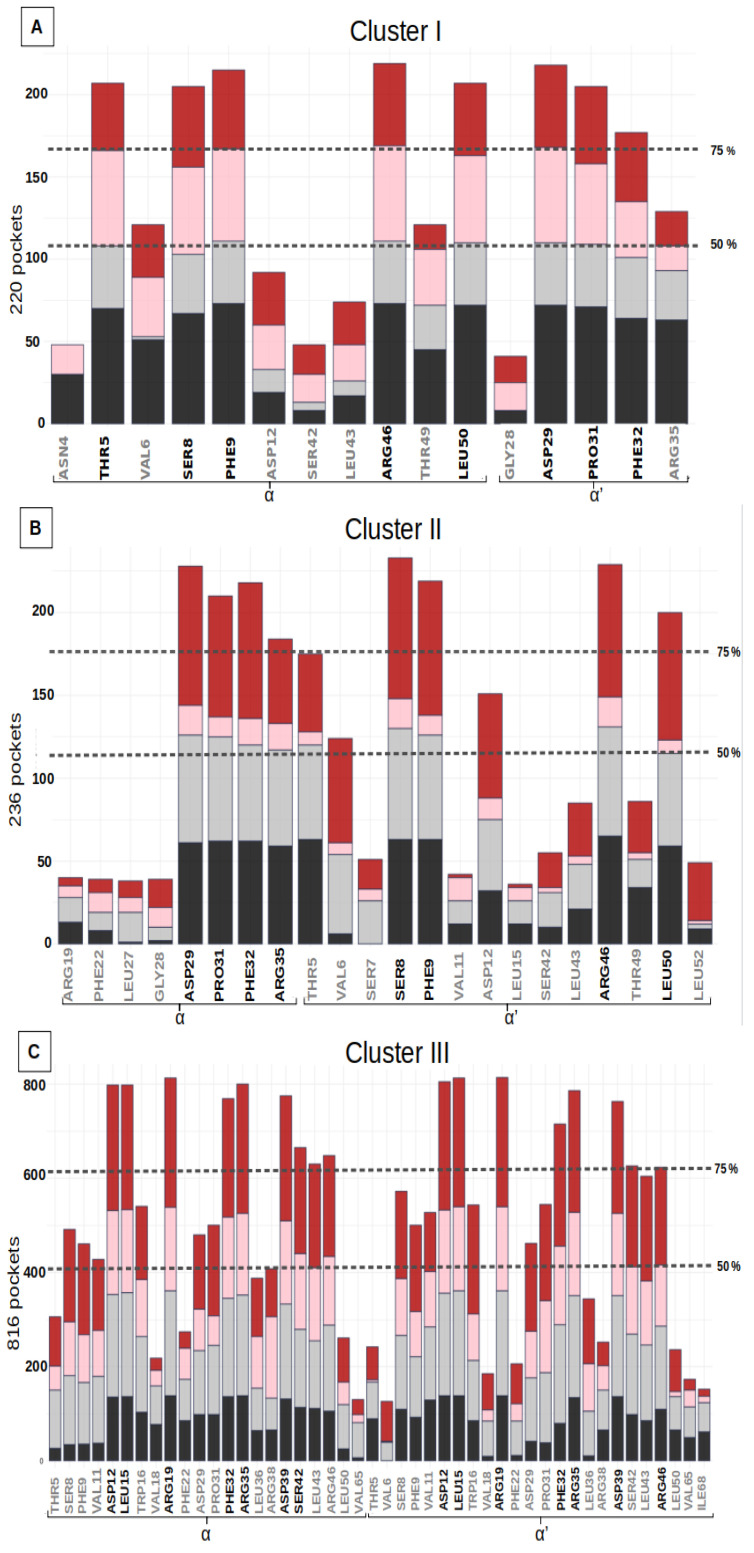
Residue frequency histograms of the druggable groove-pockets (for residues in more than 15% of the pockets) for the three clusters: (**A**) Cluster I, (**B**) Cluster II, (**C**) Cluster III. The bars in the histograms represent the distribution of the number of pockets, including a specific residue for each of the structures H6N6_c, H6N6_so_o, H1N1_so_c, H5N1_o shown in black, gray, pink and red respectively. Two dotted lines indicate the residues seen in more than 50% and 75% of the pockets in the class studied, and the residues selected as key residues in each class are shown in black on the X axis.

The variability analysis of the residue of the pocket cluster allows for the study of the deformability of the site. The most frequent residues of the pocket cluster characterized the key residues of the binding site. The residue frequency of the three pocket clusters is summarized in histograms, with the proportion of the four initial structures indicated in Figure 3. Each binding site includes stable and frequent residues, observed in 75% of the pocket cluster. These frequent residues are considered as key residues that critically determine the druggability of the binding site. Each pocket cluster also includes some residues that are much less frequently observed (<25% of the pockets cluster). These residues appear anecdotally in the binding site, when we consider its deformability along the simulations. The three sites were regularly observed in the MD simulations of the four different structures (Figure S3). These analyses confirm that not only the key residues, but also most of the associated residues, are observed on the four different structures. The key-residues of the binding sites are listed in Table 2.

**Figure 4:**
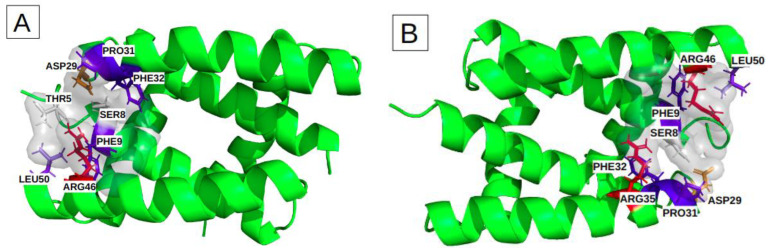

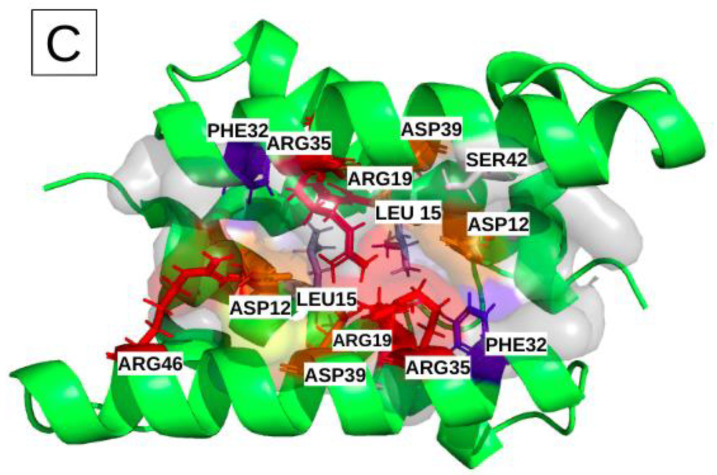
Characterization of the physico-chemical properties of the three druggable groove-pockets identified according to the classification into three clusters: the most frequent residues are colored in red and orange for the positively and negatively charged ones, in blue and slate-blue for the aromatic and aliphatic ones, and in white for the polar. (**A**) is cluster I, which is a small pocket at the end of the groove, (**B**) is cluster II, which is a small pocket located at the opposite side of cluster I. (**C**) is a large pocket identified on cluster III, located in the center of the groove, recovering a large groove interface.

Binding sites I and II include height key-residues. The height key-residues of site I are shown in Figure 4A. Binding site II exhibits eights similar key-residues, but on the opposite chains (Figure 4B). These two sites are located at the two opposite extremities of the groove. The presence of three aromatic or aliphatic key-residues (PHE, PRO and LEU) likely accounts for the high druggability score of their relatively small pockets. We also notice that these sites involve two polar amino acids (THR, SER) and two charged key-residues (ARG and ASP) (Table 2).

The most frequent binding site, III, corresponds to larger pockets (31 residues on average) that involve 14 key residues. Their corresponding pockets are symmetrical, according to the alpha helices 2 and 2′, and they are located in the center of the groove (Figure 4C). The high druggability score of 71% ± 13 likely relies on (i) its large size, (ii) the presence of two aromatic (PHE) and two aliphatic (LEU) residues and (iii) one polar amino acid (SER). We noticed also the presence of nine charged key-residues (ARG and ASP) bordering the pockets.

### 3.4. Identification of a Large Common Binding Site

We then set out to further analyze binding site III. Because of its large size, its accessibility and its central position in the middle of the groove, along with its high druggability, this binding site appears the most suitable one to target with a drug-design approach. A small compound occupying this site is likely to prevent or to disrupt the NS1s interaction with RNAs. This site III very frequently adopts a druggable conformation, and involves a large number of residues, including the 14 key residues listed in Table 2.

**Figure 5:**
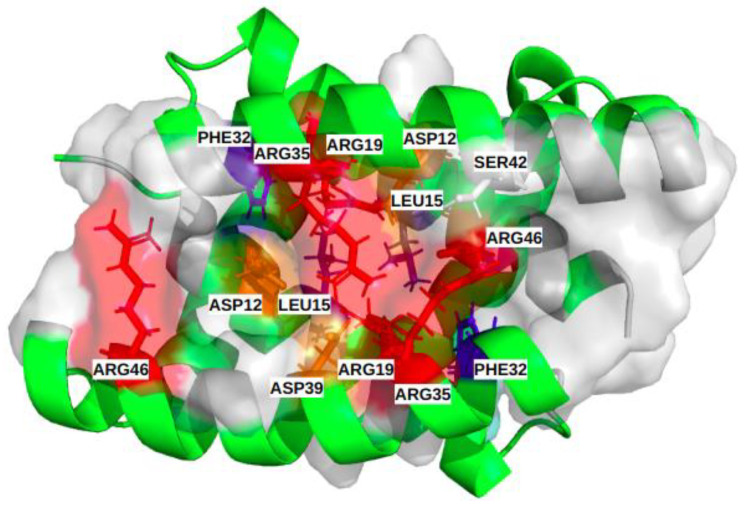
Illustration of the common binding site of the different subtypes, including the 14 most frequent residues (α [ASP12, LEU15, ARG19, PHE32, ARG35, ASP39, SER42); α’ [ASP12, LEU15, ARG19, PHE32, ARG35, ASP39, ARG46]). Physico-chemical properties of the corresponding residues colored red and orange for the positively and negatively charged ones, respectively, blue and slate-blue for the aromatic and aliphatic ones, respectively, and white for polar.

It is generally recognized that pockets with at least 14 residues, and exceeding the volume of 600 Å^3^, are the most suitable for binding drug-like molecules [36]. Based on this rationale, we decided to deepen our analysis on the pockets of cluster III, which contain simultaneously 14 key residues. This results in a sub-cluster of 424 pockets, observed in more than 38.3% of the 1102 druggable RBD-grooves (i.e., in 21.2% of the 2000 time-sampled conformations). This sub-cluster of pockets is found in the different subtypes with a similar distribution: 21.8%, 27.5%, 26.4% and 33.7% for the four forms, respectively. The corresponding binding site is called a “common binding site” (Figure 5).

Figure S1 shows the hierarchical classification of the heatmap of the 424 “common binding site” pockets, in the MD simulations with information on the residues involved and the respective extracted groove-pocket numbers. The supplementary band with four colors (black, grey, pink and red) indicates the structures (H6N6_c, H6N6_so_o, H1N1_so_c and H5N1_o, respectively), from which the pockets are extracted.

The preference of some moderately frequent residues for one of the four structures can be noted. For example, residues ASN4, PHE22, ASP24, GLN25, GLU26, LEU27, GLY29 are predominantly observed in pockets associated with the semi-open form with close tendency (H1N1_so_c). The residues PHE14, ASP34, ILE68, LEU69 are mainly observed for pockets associated with the H6N6_so_o and H5N1_o forms.

Finally, this analysis confirms the existence of a broad-consensus druggable binding site (with a common base of 14 key-residues), present in all viral subtypes and whatever the NS1 forms and the length of the linker. Its associated pockets tend to be polar, charged, moderately hydrophobic and composed of a large number of residues.

## 4. Discussion

In this study, we focused on the selection and characterization of pockets on the RNA-binding domain of the influenza A protein NS1, using conformations that were time-sampled during four parallel 150 ns molecular dynamics simulations from four distinct initial structures of full-length NS1. The application of unsupervised methods such as the hierarchical clustering of pockets allowed for the identification of the main druggable binding sites of the groove region. The study of binding-site flexibility and its variability in terms of residues allow for the description of its deformability during MD simulations for the different forms of NS1. It confirms that, despite the stability of the RBD, the MD simulation induces local movements of the side chains of the residues involved in the groove region, and impacts the RNA binding site. Indeed, MD simulations, by probing the flexibility of the initial crystal structures, have revealed the druggability of the groove-pockets which were below the druggability threshold in three of the four static crystallographic structures used in this study [37].

We identified, through the hierarchical classification of the pockets according to their composition in terms of residues, three main pocket clusters. Two of these (binding sites I and II) are rather small, and less frequently in a druggable conformation. Sites I and II are located at opposite extremities of the groove formed by the α2 and α2′ helices. In contrast, binding site III is much larger, and much more frequently in a druggable conformation. This pocket, at the center of the groove, could be used as a drug binding site that is able to host a wider variety of ligands, and would be more likely to prevent RNA-RBD interaction. MD simulations of the four structures (H6N6_c, H6N6_so_o, H1N1_so_c, H5N1_o) revealed that this central pocket frequently adopted a druggable conformation, thanks to the involvement of its 14 most frequent residues. This accessible central pocket, which is shared by the four structures, has a high therapeutic potential. This common pocket could be targeted by drug-design approaches in order to identify small compounds that may prevent the interaction of NS1 with RNAs, and thereby inhibit its activities, regardless of sequence variations of the protein.

In some protein–ligand complexes, the variation has been shown to correlate with the electrochemical characteristic of the ligand molecules. To achieve binding, proteins have been observed to engage in subtle balances between electrostatic and hydrophobic interactions, to generate stabilizing binding-free-energies [38]. Physico-chemical complementarity is generally considered to be the driving force behind molecular bonding. Complementarity of electrostatic potentials, for example, is considered to be the force that attracts the ligand from the solvent to the binding site [39]. The hydrophobicity of the binding pocket is generally correlated with the properties of the ligands in protein–ligand complexes. The hydrophobic parts of the ligand often interact with the hydrophobic parts of the protein [40]. Looking more closely at the physico-chemical properties of the groove-pockets identified at the RBD–RNA interface, we noticed the presence of several positively and negatively charged residues, such as (ARG19, ARG35, ARG46) and (ASP12, ASP29, ASP39). The presence of charged residues promotes the drug-like molecules binding in the pockets, mainly through the formation of hydrogen bonds or salt bridges. For example, arginine residues play a role in partially neutralizing the ligand charges in the binding pocket, allowing ligand to enter. Positive electrostatic patches above and below the binding entry also contribute to the main attractive forces that lead the drug to the protein surface near the binding site [41].

Hydrophobic residues are also identified (PHE9, LEU15, PRO31, PHE32, LEU50) as well as a few polar residues (THR5, SER8, SER42). Studies have shown that there is a high correlation between the hydrophobicity of the molecular environments and the experimentally determined desolvation energies. Similar to the cores of proteins, the binding sites of small molecules are often made of hydrophobic residues that could positively contribute to the binding of organic molecules in aqueous environments, and are involved in maintaining a stable 3D arrangement of the ligand in the binding pocket [42,43,44].

The polar and positively charged amino acids such as SER and ARG, which form strong ionic hydrogen bonds (salt bridges), are known to be the most favorable contacts for protein–RNA interaction [45,46,47]. Recently, Wacquiez et al., 2020, in their study of the NS1 structure and sequence determinants governing the interactions of RNAs in the case of H7N1 (PDB: 6SX2), described how the RBD-groove aligns itself almost perfectly in parallel to the RNA, to bind it. These authors underlined several RBD-groove residues involved in the RNA interaction [48]. Our three identified binding-sites include six residues (THR5, ASP29, PRO31, ARG35, SER42, ARG46) of the ten described by Cheng et al., 2008, (THR5, ASP29, ASP34, ARG35, ARG38, SER42, ARG46, THR49), as directly involved in the interaction with RNA; the limitation of their study lies in the fact that they did not take into account the NS1 polymorphism [49]. Our study confirms the presence of this binding site in the different strains, while taking into account the NS1 polymorphism. Five other contacts were described by Wacquiez et al., 2020, (PRO31, ARG35, ARG37, ARG38, THR49). Two of these residues, ARG38 and THR49, also detected in the RNA interactions, are partly observed (in approximately 50% of cases) in the pocket clusters of binding sites I and III.

Although the three binding sites are generally identified separately on the conformations, we were able to observe the presence of the three pockets simultaneously on a single conformation (Figure S2). The three pockets can therefore coexist, but in a rarer way (0.7%), revealing a much larger interaction interface with RNA.

The results of our work, as well as the literature elements, confirm our hypothesis that the RNA binding domain of the NS1 protein is a promising therapeutic target, firstly because the frequent druggable pockets identified at the RBD interface show favorable physico-chemical and geometrical properties for hosting a drug candidate, and secondly because this druggable region is identified in the different subtypes (H6N6, H1N1 and H5N1) of the influenza A virus.

## 5. Conclusions

Our study shows the relevance of combining molecular dynamics simulations with the monitoring of pockets and the prediction of their druggability. Indeed, with this approach, we were able to identify three well-characterized binding sites in the groove region of the NS1 RBD and describe their variability. Unsupervised classification allowed us to confirm the presence of these binding sites across the four distinct structures representing three sequence variants of NS1. We also noticed that the key residues involved in the high druggability scores were highly or strictly conserved across the four NS1s from the subtypes H6N6, H1N1 and H5N1.

In this paper we worked on four full-length structures of the NS1 protein of the influenza A virus. These four structures were co-crystallized in different forms (closed, semi-open with closed tendency, semi-open with open tendency, and open shape). In our previous study, we were able to demonstrate that the RBD of the NS1 protein was highly stable during molecular dynamics simulations, regardless of the initial form of the structure. Our work therefore consists of searching for protein cavities likely to host a drug candidate capable of inhibiting the interaction between the RBD domain of the NS1 protein and RNA on these different structures and subtypes. In order to verify whether there is a druggable binding site on their surface, and what its properties would be, we coupled molecular dynamics simulations, pocket search, druggability prediction, and statistical approaches.

According to our previous study in Naceri et al., 2022, NS1 can adopt its various forms, regardless of its sequence that characterize its plasticity. Here, we were also able to extract three main druggable binding sites located at the groove (between α-helices 2 and 2′) on all four structures. More than 55% of the groove is druggable, whatever the structural polymorphism. The four structures we have identified have a large binding site in the center of the groove that is highly druggable and involves 14 key residues, some of them being directly involved in RNA interaction. In more than 20% of the time-sampled conformations in the MD simulations, this site has a high druggability score. This confirms the NS1 RBD groove as a good therapeutic target, as it would be able to host a universal therapy effective on the different subtypes.

## Figures and Tables

**Table 1:** Number of groove-pockets (total or druggable) and groove conformations, according to the four forms of NS1 (H6N6_c, H6N6_so_o, H1N1_so_c, H5N1_o) obtained in the initial static PDB structures and on a set of 500 conformations sampled during the MD simulations.

Subtype_Forms	H6N6_c	H6N6_so_o	H1N1_so_c	H5N1_o	Total of Four Structures
**Initial (static) structure/MD sampling conformations**	1/ 500	1/ 500	1/ 500	1/ 500	4/ 2000
**Number of groove-pockets [Initial structure/** **MD conformations (%)]**	2/ 933 (27.7)	1/ 861 (25.6)	3/ 740 (22.0)	2/ 826 (24.5)	8/ 3360
**Number of druggable groove-pockets ** **[Initial structure/MD conformations (%)]**	0/ 276 (29.6)	0/ 329 (38.2)	2/ 257 (34.7)	0/ 410 (49.6)	2/ 1272 (37.9)
**Occurrence of druggable groove conformations ** **[Initial structure/** **MD conformations (%)]**	0/ 239 (47.8)	0/ 285 (57.0)	1/ 233 (46.6)	0/ 345 (69.0)	1/ 1102 (55.1)

**Table 2:** Table of the three groove-druggable binding sites (I, II, III). Total pocket number observed in different clusters or binding sites. Average druggability score of pockets associated with each binding site and its corresponding standard deviation (sd). Key residues observed in more than 75% of the cluster of druggable pockets for each binding site are listed.

Pocket Clusters	Pocket Number	Druggability Score Mean (sd)	Key Residues of the Clusters
**I**	220	0.66 (0.12)	8 residues: α: THR5, SER8, PHE9, ARG46, LEU50 α’: ASP29, PRO31, PHE32
**II**	236	0.68 (0.13)	8 residues: α: ASP29, PRO31, PHE32, ARG35, α’: SER8, PHE9, ARG46, LEU50
**III**	816	0.71 (0.13)	14 residues: α: ASP12, LEU15, ARG19, PHE32, ARG35, ASP39, SER42 α’: ASP12, LEU15, ARG19, PHE32, ARG35, ASP39, ARG46
**Total**	1272	0.68 (0.12)	

## Data Availability

Data are contained within the article.

## Supplementary Materials

The following supporting information can be downloaded at: https://www.mdpi.com/article/10.3390/biom13010064/s1. Table S1. Summary of the four structures (H6N6_c, H6N6_so_o, H1N1_so_c, H5N1_o), corresponding Uniprot (Q20NS3, P03496, A5A5U1) and PDB Identifiers (4OPA, 4OPH, 5NT2, 3F5T) together with information of forms (close, semi-open with close tendency/open tendency and open), linker size, and details of reversed mutations in the homology modeling Figure S1. Heatmap of the 424 common binding-site pockets of the four structures H6N6_c, H6N6_so_o, H1N1_so_c and H5N1_o colored in black, gray, pink and red respectively. The Y axis corresponds to the list of residues that compose the pockets in order of appearance from top to bottom on the two chains, in succession. Their 14 key residues are indicated with cyan arrows on the Y axis enclosed by the chains α and α’ (α [ASP12, LEU15, ARG19, PHE32, ARG35, ASP39, SER42); α’ [ASP12, LEU15, ARG19, PHE32, ARG35, ASP39, ARG46]). The X axis lists the druggable groove-pockets clustered according to their similarity in terms of residue composition. Each column represents a pocket and the residues it contains. Figure S2. Representation of one rare case of NS1 conformation (conformation 133 of 4OPA MD simulations) that visits simultaneously the three identified groove binding sites. The blue pocket corresponds to binding site I, the orange to binding site II and the yellow to binding site III. (A) Full-length representation (B) RBD representation, (C) Coloring of one pocket representative of each of the three clusters colored according to the chemical nature of its amino acids: positively charged, negatively charged, polar and hydrophobic amino acids are respectively indicated in in red, blue, white and purple. Figure S3. Dotplot of three identified clusters (Y axis) observed per conformation for a set of 100 conformations (X axis) sample on molecular dynamics simulations of the four structures, H6N6_c, H6N6_so_o, H1N1_so_c and H5N1_o. For each conformation (X axis), the presence of a pocket belonging to clusters I, II and III is indicated by a blue, orange and yellow square, respectively.
