# Supplementary material for: Druggable Pockets at the RNA Interface Region of Influenza A Virus NS1 Protein Are Conserved across Sequence Variants from Distinct Subtypes
Source: Biomolecules. 2022 Dec 29;13(1):64. doi: 10.3390/biom13010064 (PMC9855689; doi:10.3390/biom13010064)
Supplement: Supplementary file 1 [file biomolecules-13-00064-s001.zip › biomolecules-2107000-supplementary.pdf]

biomolecules

MDPI

Supplementary Materials:

Table S1. Summary of the four structures (H6N6_c, H6N6_so_o, H1N1_so_c, H5N1_o), corresponding Uniprot (Q20NS3, P03496, A5A5U1) and PDB Identifiers (4OPA, 4OPH, 5NT2, 3F5T) together with information of forms (Close, Semi-open with close tendency/open tendency and Open), linker size, and details of reversed mutations in the homology modeling

**Structures**	IDs	Forms	Reverse mutations details
H6N6_c	Uniprot: **Q20NS3** Xray PDB: 4OPA	Close (dED-ED= 27.8Å) Short linker (Δ 80-84)	R38A/K41A
H6N6_so_o	Uniprot: **Q20NS3** Xray PDB: 4OPH	Semi-open with open tendency (dED-ED=60.0Å) Long linker	R38A/K41A
H1N1_so_c	Uniprot: **P03496** Xray PDB: 5NT2	Semi-open with close tendency (dED-ED= 32.4Å) Long linker	R38A/K41A/W187A
H5N1_o	Uniprot: **A5A5U1** Xray PDB: 3F5T	Close (dED-ED= 66.5Å) Short linker (Δ 80-84)	R38A/K41A

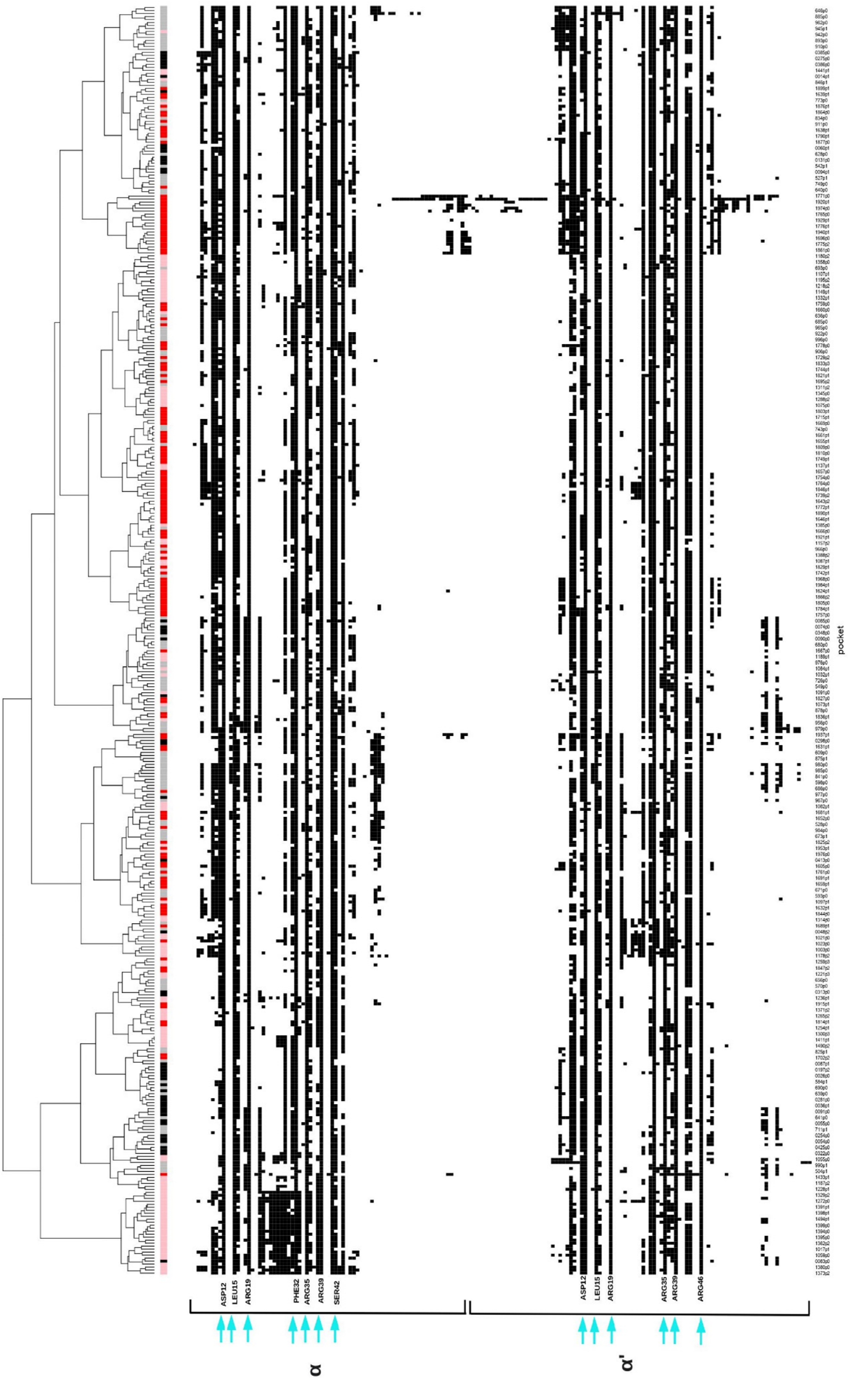
ASP12
LEU15
ARG19
PHE32
ARG35
ARG39
SER42
α
ASP12
LEU15
ARG19
ARG35
ARG39
ARG46
α'
pocket

Figure S1. Heatmap of the 424 common binding site pockets of the four structures H6N6_c, H6N6_so_o, H1N1_so_c and H5N1_o colored in black, gray, pink and red respectively. The Y axis corresponds to the list of residues that compose the pockets in order of appearance from top to bottom on the two chains in succession. Their 14 key-residues are indicated with cyan arrows on the Y axis enclosed by the chains α and α' (α [ASP12, LEU15, ARG19, PHE32, ARG35, ASP39, SER42); α' [ASP12, LEU15, ARG19, PHE32, ARG35, ASP39, ARG46]). The X axis lists the druggable groove-pockets clustered according to their similarity in terms of residue composition. Each column represents a pocket and the residues it contains.

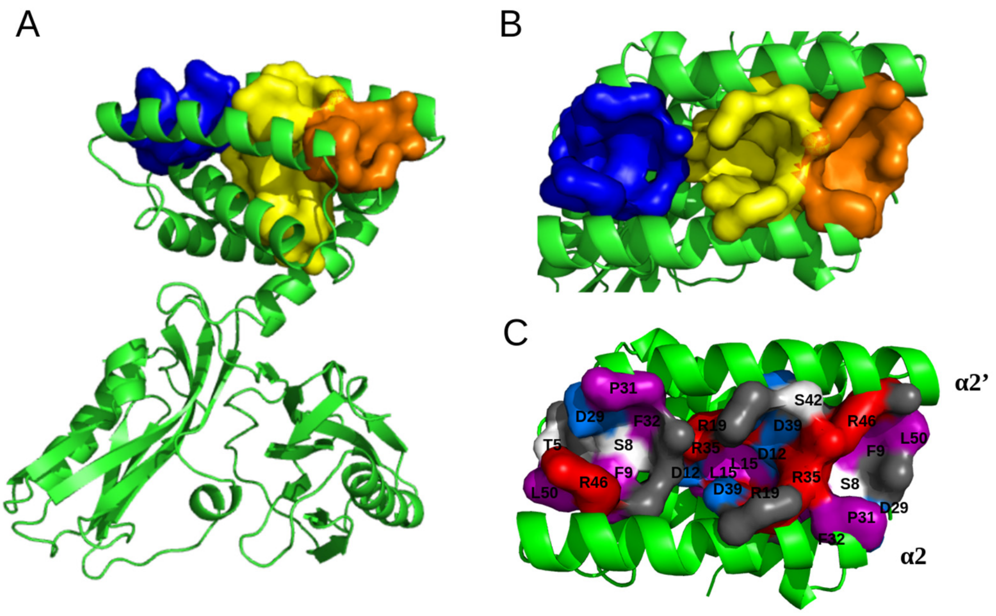

Figure S2. Representation of one rare case of NS1 conformation (conformation 133 of 4OPA MD simulations) that visits simultaneously the three identified groove binding sites. The blue pocket corresponds to the binding site I, the orange to the binding site II and the yellow to the binding site III. (A) Full length representation (B) RBD representation **(C)** Coloring of one pocket representative of each of the three clusters colored by chemical nature of its amino acids: positively charged, negatively charged , polar and hydrophobic amino acids are respectively indicated in in red, blue, white and purple.

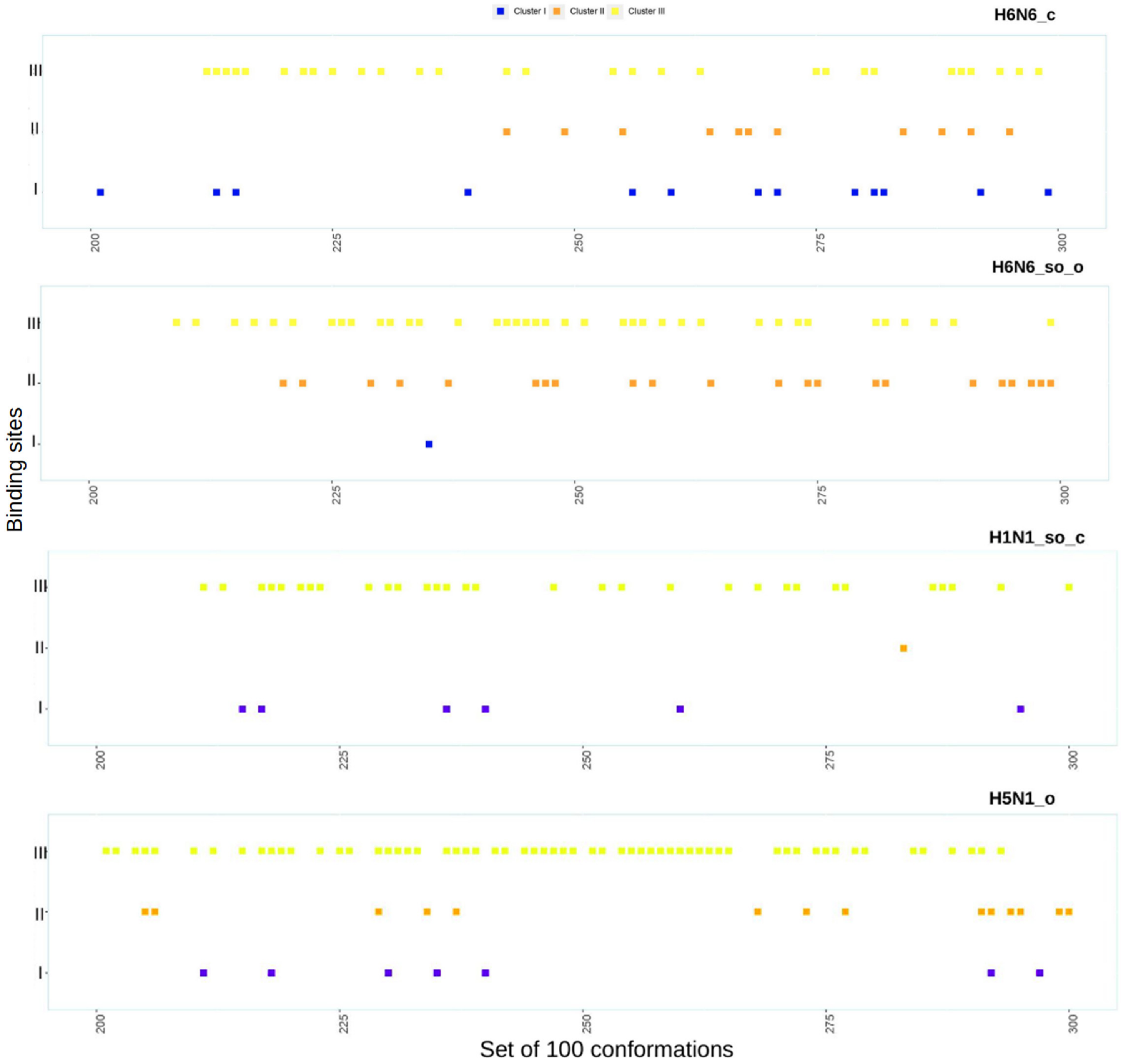

Figure S3. Dotplot of three identified clusters (Y axis) observed per conformation for a set of 100 conformations (X axis) sample on molecular dynamics simulations of the four structures H6N6_c, H6N6_so_o, H1N1_so_c and H5N1_o. For each conformation (X axis), the presence of a pocket belonging to clusters I, II and III is indicated by a respectively blue, orange and yellow square.
